# The Hospital Nursing Supplement

**Published:** 1894-06-09

**Authors:** 


*The Hospital*, June 9, 1894. *Extra Supplement.*

"The Hospital" Nursing Mirror.

Being the Extra Nursing Supplement of "The Hospital" Newspaper.

[Contributions for this Supplement should be addressed to the Editor, The Hospital, 428, Strand, London, W.C., and should have the word "Nursing" plainly written in left-hand top corner of the envelope.]

### News from the Nursing World.

### HONOURABLE OBLIGATIONS.

"A nurse is expected always to wear uniform when with a case," is amongst the rules at most institutions undertaking to supply private nurses. It appears one that should certainly never be questioned, and perhaps it seldom is—verbally, but it is evaded every day. There seems a curious absence of honour in this matter, for the nurse who receives her last intructions from her superintendent, in a dress admirably suited to her professional position, does not hesitate, when she is safely established amongst strangers, to exchange the well-fitting print dress for a skirt and a "blouse." Whatever the colour of the latter—and even scarlet is somewhat donned—the style is always untidy, and as for the excuse that the garment is "so nice to work in," it carries little weight because far more actual manual labour is hourly accomplished by the trim ward nurse in her pretty and suitable uniform than often falls to the lot of her sister in a private family. Blouses or shirts are suitable and fashionable in the proper place, but they are neither when seen on the shoulders of the nurse who wilfully disregards the rules of her institution.

### PRINCESS CHRISTIAN AT SOHO.

The new buildings at the Hospital for Women, Soho Square, were formally opened on June 1st by Princess Christian. The out-patient hall was utilised on the occasion, and was well filled with guests when the ceremony took place. The Duke and Duchess of Westminster, the Duchess of Buckingham, and Lady Romilly were amongst those present, and purses containing donations to the Building Improvement Fund were graciously received by Her Royal Highness. Besides the new out-patient department a number of separate bed-rooms for the nursing staff have been erected. Each has a fire-place, and is neatly furnished with spring bed, washstand and chest of drawers, and looking-glass fastened on the wall. Various additional fittings are contemplated which will make the little rooms very comfortable. The sanitary arrangements appear good, and the nurses' bath-room, with its tiled floor and walls, is excellent.

### A PRIZE QUILT.

A prize competition for a patchwork quilt has just earned £17 for the Swanage Cottage Hospital. The successful quilt was designed in four divisions, each representing a horse-shoe, and consisting of eighty squares of white linen, worked in blue, in a variety of stitches. The quilt was raffled for, and secured the sum mentioned.

### AN ENGLISH HEROINE.

The testimony to courageous Nurse Hinton, who in the face of much opposition has effectually championed the cause of the sick poor at Newton Abbot, continues to grow, and it is now approaching the sum of £100, which the promoters desire to collect. Amongst the most touching contributions may be reckoned the pence received from poor villageıs, and the collection made by the seamen and marines of H.M.S. *Cambridge*.

### NEWTON ABBOT.

The official inquiry into the management of Newton Abbot Workhouse is now being conducted in a thoroughly satisfactory manner, inasmuch as the investigations will go back over ten years. The Guardian who, according to the local press, offered to spend five pounds in proving Nurse Hinton had perjured herself, must be surprised to find that her statements are likely to show as extremely moderate ones. When the present master was appointed at Newton Abbot Workhouse in 1884 he reports that there were nearly 300 paupers in the house, 125 of whom were old and infirm inmates of the sick wards, 16 were imbeciles, 36 temporarily disabled, and 15 infants. There was only one nurse, a woman of sixty-five, and the suggestion made in 1890 by a medical officer that two nurses should be appointed, was not carried out. In fact, the discrediting, not the reforming, of reported evils appears to have been hitherto the general policy.

### "REGISTERED" NURSES.

Under the designation of "The Registered Nurses' Society" an association has been recently started, with offices in Regent Street. Doubtless there may be room for a new nurses' agency, established on the same principles as the Nurses' Co-operation, which has steadily prospered and increased at 8, New Cavendish Street; but a somewhat illogical deduction on the subject has found its way into the press. This is that as the nurses of this new society will be all on the List (erroneously called a register) of the R.B.N.A., therefore "an assurance is given to the public that every nurse provided by it has had at least three years' hospital training." Now, it was proved conclusively before the Privy Council that many of the earlier members who joined this association had not had the three years "hospital training" at present required. And further, at a recent election of members, one nurse elected had obtained her certificate at Buchanan's Cottage Hospital, where the nursing staff consists of two nurses and two probationers, and the number of beds is about twenty. We therefore rely upon the press in the interests of the general public to make it widely known that a "registered" nurse may have trained only in a cottage or other special hospital; and that there are, in fact, no such things as "registered" nurses in any proper meaning of that term. Hence some at least of the nurses of the new society cannot fairly be accredited with holding so high a professional position as the many women who have gone through two or three years' complete training in the large school of a general hospital or infirmary, and who may have declined to become members of the Royal British Nurses' Association. It follows that the public derive no definite guarantee from this so-called "Register," which can only claim at best to be what it in fact is—a list of the members of one comparatively small association.

### POOR AND HELPLESS.

The cruelties alleged to have been practised on the children in Hackney Training School by Ella Gillespie are being inquired into, and it is probable that the Local Government Board will take up the matter of the management of these schools. The ill-treatment to which the helpless little paupers are said to have been subjected for years at the hands of one woman is a very strong argument for the proper supervision of all Poor Law schools. Signs of the blows and other tortures inflicted on tender little bodies must have been only too visible, and therefore it is impossible to imagine how these cruelties can have escaped detection from some of those persons who are responsible for the proper conduct of state-supported schools.

### CARETAKERS FOR IDIOTS.

It appears that the Local Government Board issues a "query" sheet as to the qualifications and antecedents of nurses who are to be employed in Irish workhouses, but according to the local press, these questions cannot always be satisfactorily answered. Still, it is strange to find the Tobercurry Guardians discussing the possibility of one of the inmates being able to fill the responsible post of temporary nurse in the idiot ward after confessing that a person they had already chosen was not up to the Local Government Board standard. If any inmate is found to possess all due mental and physical qualifications for intelligent nursing, it is natural that a question should arise as to why such a competent woman is satisfied to remain an inmate of a workhouse!

### NEW RULES AT ADDENBROOKE'S HOSPITAL.

The new rules framed for the nursing staff at Addenbrooke's Hospital seem to be, on the whole, judicious ones, although, perhaps, a little more careful revision of the wording in places might have tended to clearness. The duration of holidays and the hours off duty are not stated, which is certainly a regrettable omission. We read: "The members of the nursing staff shall be appointed by the weekly board on the recommendation of the matron. They may be dismissed by the weekly board." Surely these phrases convey a false impression. The matron should certainly have a voice in discharging as well as in engaging the nursing staff, although in both cases she should naturally be associated with the board, and not act independently of it. The special rules which deal with the personal duties of the nurses are sufficiently definite, and they provide for the efficient training of probationers and for the guidance of the rising generation of nurses who may not be as well-informed as their older sisters on points which have become established observances in most of our hospitals.

### POPULAR APPOINTMENTS.

Considerable satisfaction has been expressed in Scotland at the appointment of Miss Græme as Superintendent of the Perth Sick Poor Nursing Association, and of Miss M'Queen as nurse. The connection of both these Scotch ladies with Perthshire is regarded as a subject for cordial congratulation in the district, and certainly the Queen's Nurses seem to be highly appreciated in the north.

### BATHS IN ASYLUMS.

The subject of baths for lunatics is getting a good deal of consideration in Paris just now in consequence of the accidental scalding of an insane patient. One member of the committee which has been appointed to go into the matter of legislation for the insane is reported to have said that the hot water should be poured in after the patient is placed in the bath. This plan hardly seems feasible, and most trained male or female nurses would hesitate before undertaking so perilous a task. Putting hot water into a bath where a sane person is established requires very great care, and certainly in the case of a lunatic would present even greater difficulties.

### NURSING AT HAMBURG.

A correspondent to a contemporary draws a not very alluring picture of the condition of nursing in Germany. At the Eppendorfer Hospital, Hamburg, the majority of the nurses are comparatively uneducated, and usually take a lower position than their professional sisters in England and America. The probationer who has been accepted and has successfully passed the medical examination, has sometimes months to wait before a vacancy on the staff allows her to be placed on duty, and in the interval "the probationer plays charwoman." The course of instruction is not as helpful as it might be. The average pay in private nursing, judged by our standards, is very poor, ranging only from fifteen to thirty-five marks a week.

### SICK NURSES IN DENMARK.

According to the latest statistics, there are at present in Denmark, outside Copenhagen, 129 societies with 171 nurses, of which societies 51 are in towns and 78 in the country. In 1883 there were altogether 37 societies with 58 nurses, and in 1888, 77 societies with 111 nurses. In many of the societies the nursing is entirely gratis, and in them all there is free nursing, or nursing at much reduced prices to poor patients. In many of the societies money and other help are given to poor patients, and appliances are lent. Several corporations and also various private people find board and lodging free of charge for sick nurses; and in several of the societies funds are being collected for establishing an old age pension institution for sick nurses.

### SHORT ITEMS.

*Nursing Notes* for June contains many excellent articles on nursing subjects.—The Princess Christian has consented to receive the gold medal of the National Health Society.—The Wolverhampton Queen Victoria Nursing Association held its annual meeting recently and gave a satisfactory report of its work. The institution was started as a local commemoration of the Queen's Jubilee, and it has now a staff of 32, 25 of these being private nurses.—It has been decided to erect a Nurses' Home for the staff of Worcester Infirmary in Whalley Gardens, which are quite near to the institution.—At the recent fire in Wright Street, Hull, some fine oak panelling as well as the flooring of an upstairs room at the Nurses' Home was considerbly damaged.—Miss Blakemore, of Mrs. Mackalpine's Maternity Home, Webster Street, Greenheys, Manchester, was successful in obtaining the diploma of the London Obstetrical Society at the recent examination; she was prepared for this examination by the honorary medical attendant of the Home.

### On General Nursing.

By Rowland Humphreys, M.R.C.S., L.R.C.P.Lond.

XV.—RHEUMATISM (*continued*).

Very early in the disease, often before the case is seen for the first time, the heart becomes affected. The inflammation of the heart may be either of its interior or exterior. In the former case it is called endocarditis, in the latter pericarditis. *Endocarditis* is an inflammation of the lining membrane of the heart, which commences in the neighbourhood of a valve, and thence spreads to the surrounding structures. It is of importance both for its immediate and for its remote effects. The immediate effect lies in the implication of the cardiac muscle, and in the danger of a failure of the circulation from the valves not acting properly; the remote danger is that the valves may be puckered or the apertures they cover contracted or dilated by the contraction of the inflammatory material, and thus the valves may be insufficient to close the apertures, or these last may be too small to permit the requisite flow of blood through them at each beat of the heart. The result of heart disease is always the same—that the blood fails to be driven forward as fast as it should, and tends to remain in the lungs, liver, and vessels of abdomen and lower extremities. Sometimes the condition of the heart is best shown by the size of the liver. In cases of sudden failure of the heart the liver becomes over distended and enlarged, and the patient complains of pain and of tenderness or pressure below the ribs on the right side. As the condition of the heart improves, under rest and medicinal treatment, the liver gets smaller, and the tenderness and pain pass off.

The symptoms of endocarditis are those of uneasiness or pain in the region of the heart, the pulse is usually quickened in a well marked manner, and weak, and there may be some pain on taking a deep breath. The symptoms of this complaint are not so well marked as those of pericarditis, especially with respect to pain. On listening over the heart the clear-sounding tone characteristic of the normal sounds of the heart is absent, and the sound either prolonged, or muffled, or rough. This alteration in character is indicative of some roughening or alteration in shape of the affected valve. The valve which is inflamed is known by the alteration in the heart's sounds, both in relation to one another and to their position on the chest wall, where best heard. Thus if the mitral orifice be dilated the abnormal sounds are heard at the apex of the heart, in the axilla, and often behind, at the angle of the scapula. If the aortic valves were so injured that they allowed the blood to return into the ventricle the rough sound would be heard at the apex, and hardly, if at all, at the angle of the scapula or in the axilla. Then if the sound indicative of disease be heard in company with the first sound following or prolonging it, it means either that the ventricle, in contracting, forces its contents back through the over-large or insufficiently guarded mitral orifice, or that the aortic orifice is too small, according to the valve indicated, as the sound itself is due to the contraction of the ventricle. If the second sound is prolonged, it indicates that the aortic orifice is too large or is misshapen, and that the blood, instead of flowing steadily on into the general circulation on the closure of the valves, is being forced by the elastic contraction of the aorta, amongst other things, back into the ventricle, there dilating and irritating the heart. Other diseases of the valves of the heart are diagnosed in a similar manner. The principal thing is to find out the condition of the heart's muscle, for if that is good it requires a very serious lesion of the valves to allow the circulation to come to a stop. Unfortunately, the means at our disposal for the determination of this point are very imperfect.

All we can do in cases of endocarditis is to give the heart as much rest as possible. This is accomplished by lengthening the interval of rest which occurs between each double beat of the heart by administering digitalis or other drugs which have the requisite property. Of course, the patient is kept on his back, so as to remove all unnecessary strain from the heart, and the diet is also calculated to the same end. If there is constipation an additional strain, apart from the action of the bowels, is thrown on the heart; so much so, that it is quite possible to diagnose constipation of a few days' standing from signs of tension about the heart's sounds. The author of this paper has seen a case of endocarditis occur in acute rheumatism, which he attributed to neglect in this direction.

*Pericarditis* is another of the complications of rheumatic fever. It is far more dangerous in its immediate effects than endocarditis, but less troublesome in its later results. The signs of pericarditis are usually well marked, differing in this from endocarditis. They vary with the severity of the attack, but often begin with a rigor and a rise in the temperature. Pain in the chest is generally present, increased on deep respiration, or on pressure over the heart. The respiration and the pulse rate are increased, the heart's action becoming either rapid and vigorous or violent and irregular. There is often some difficulty in swallowing. The patient is generally more or less anxious and restless. Instead of these symptoms, or combined with them, there may be delirium or coma, simulating meningitis very closely, and the more so that meningitis does sometimes occur in rheumatic fever. Various "nerve" and mental symptoms may also be present. Examination of the heart by auscultation shows that there is a roughened condition of the two surfaces of the pericardial sac, and a distinct bruit can be heard where they rub together. Unlike the bruits heard in endocarditis, this is very superficial, and often changes its position and character. On percussion, the area of dulness found over the heart on the chest wall is altered both in shape and dimensions. It is enlarged, especially towards the left shoulder, and its boundary in this direction, instead of being a straight line running from the nipple to the junction of the second left inter-costal cartillage with the sternum, bulges out so that it forms a curve with its prominence towards the shoulder.

The two surfaces of the pericardium—that is, the part of the membrane attached to the heart and that attached to the chest wall—rub against one another, and gradually inflammatory effusion takes place, and the sac of the pericardium, becoming distended with fluid, forms the bulge already described. With the separation of the two surfaces of the sac the rub ceases, only returning when the fluid has been in great part reabsorbed and the surfaces have again come into contact. It should be remembered that brain symptoms in rheumatism may mean pericarditis, and no pain in the chest should ever be allowed to pass unheeded.

Pericarditis is treated by rest, counter-irritation, leeching, fomentations, &c. It does not appear to be much affected by any treatment, however, and the principal aim is to relieve pain and distress, and to give as complete rest as possible.

### The Royal British Nurses' Association.

The new premises of the Royal British Nurses' Association, situated in Old Cavendish Street, were opened on May 30th by Princess Christian, the president. The Bishop of Ripon and Sir William Savory each gave a short address, the latter speaking on behalf of the general council of the association, to which the Princess replied. Before leaving, her Royal Highness went over the new rooms.

### The Mechanism of Movement.

### II.—THE SKELETON.

The foundation or support of the human body is the bony framework called the skeleton, in which we have a machine admirably adapted to the life we have to lead. Man requires considerable locomotive power for which lightness is essential, and his skeleton is so constructed that although composed of 210 separate bones it is no serious weight. He also requires gr.at variety and freedom of movement, and this his joints and the general structure of his bones secure. He also requires a framework at once strong and not easily broken, and no better material than bone can fulfil this condition.

The bones in the body are classified, according to form, as "long," as the humerus, femur, &c.; "flat" bones as those of the skull, and shoulder-blade or scapula; "short," as for example, the ribs and fingers and toes, beyond the wrist or ankle; "irregular" as the bones of the spine.

Take, for instance, such a "long" bone as the femur. From its position in the body it has to bear, directly or indirectly, the whole weight of the trunk, head, and upper limbs, and at the same time to be capable of great and varied movement. Bearing these two functions in view, let us see how they are provided for.

The femur is technically divided into "head" and "shaft." The head fits into a hollow, where, whilst firmly fixed, it is also capable of great freedom. The variety of movement of which this limb is capable may be seen any day by the performance of acrobats. It is attained by practising from an early age, and anyone could acquire equal power by similar practice. A characteristic of this and all other "long" bones is its cylindrical form, which gives it strength and resistance to strain. The same principle has been copied in engineering; wherever great strength and resistance are required the cylindrical form is chosen.

To thoroughly understand the real strength of bone it must be remembered that the pressure of any weight is distributed in two totally different directions. If we take an ordinary rafter resting on a horizontal beam, and on the apex of the rafter place a considerable weight—greater, in fact, than the rafter can support—what will be the result? The upright rafter will be "crushed," that is, the ends resting on the beam will be pushed outwards and the angle at the apex will become more and more obtuse. And the horizontal beam —what will be the effect on it? The pressure on its ends will be much increased outwards, and it will be "stretched."

Engineers in constructing bridges or buildings have in each case to make most careful calculations as to the nature of the strain to be resisted—whether a "crushing" or a "tearing" strain—and have so to arrange their materials as to best resist it. These same considerations have been beautifully provided for in our "long" bones. Careful experiments have been made as to the strength of various materials to resist strain; some have great power to resist "crushing" but not equal power to resist "stretching" strain, and *vice versâ*. But when bone was tested, it was found that in proportion to its consistency it has greater power to resist both strains than any other material.

Steel and wrought and cast iron are certainly stronger materials in one way than bone, the former considerably so; but if both rafter and beam were made of it, the rafter would resist the "crushing" strain, but the beam would come to grief. With cast iron it is the other way about. The beam would resist the strain, but the rafter would be "crushed," and wrought iron is like steel in this respect.

But when we take bone, it has almost equal power to resist both strains, and very lucky it is for our welfare that this should be so. At first sight it seems a contradiction that "long" bones requiring to be so strong to resist the strain put upon them should be hollow. It is, however, in accordance with a very important mechanical law, viz., that strength does not lie in the quantity but in the arrangement of materials.

To heap together more material than conduces to the greatest strength adds weight, not strength, and it is essential that bone should be light as well as strong, that it may accomplish its most important office—ease of locomotion.

It is well known that two pieces of the same kind of wood similar in size and shape, and to all appearance containing the same amount of material, may each be loaded with the same weight, one may bend but not break, the other snap in two. On examining the two pieces of wood the reason is evident, one piece has been cut the long way of the fibre, the wood that snapped has been cut sideways.

In a "long" bone that has been sawn through lengthwise the arrangement of the fibres composing bone can be seen more or less perfectly, according to the care with which the shreds of membrane, marrow, and other foreign materials have been cleared from it. All the fibres run lengthwise, and cross one another at right angles, enclosing minute square meshes as accurately as in the finest and most perfect lace.

This long substance has been called "spongy," but this is misleading, and does not convey any idea of the beauty and symmetry of the arrangement. A more accurate designation is "cancelous," from the Latin word *cancelli*, the bars composing lattice work. This is not the whole construction, however; the inner part of the bone is cancellous but not the outer, which in the sawn up long bone looks like ivory, and no interstices can be discovered by the naked eye. This formation of bone is aptly called "compact," and is invariably found on the outer side of the bone. There is, however, no difference in the composition of these two kinds of bone, one is simply more closely knit together than the other.

Applying again the principle that to obtain the greatest strength we must seek how most wisely to arrange our material, we must still keep clearly in view what the long bone has to do, namely, to bear vertically the weight of the body. Hence the greatest strain is on the outside of the pillar-like bone, and therefore we should look for the greatest strength on the outside, and here it is. This compact exterior layer of bone is not the same thickness all the way down. There is no marked difference, but if carefully measured it would be found to vary slightly. The variation is not haphazard, but is in accordance with another mechanical law. The bone is both hollow and cylindrical. Now the weakest point of a cylinder, and that which has also the greatest strain upon it, is not the centre, as might be supposed, but on either side of the centre. Hence, true to mechanical principle once more, the amount of compact material in the bone has been very slightly increased at these points to meet the weakness to some extent. We frequently have practical proof that these are the weakest points because fractures are very much more frequent at these than at any other parts.

### Typhoid and Maternity.

Trained nursing for the sick poor is become a generally accepted necessity, and it is to be hoped that some knowledge of general diseases will soon be extended to the guardians themselves. We trust that not many boards are as indifferent or as unconscious as the one at Cheltenham, recently reported by the local press as permitting the nursing of a bad case of typhoid fever to be done by the attendant in charge of two maternity cases. The chairman remarked at the weekly meeting, according to the *Cheltenham Examiner*, that this proceeding "was highly dangerous to mother and child, and little short of a scandal."

### News from Melbourne.

(Contributed.)

Typhoid continues to maintain an extraordinarily high rate, which puzzles the medical men, and it is even more severe in the country than in Melbourne; while a town in the smaller district has lately suffered very severely, and Romsey, in another direction, with quite different soil and surroundings, is suffering as severely from the epidemic.

With the approach of winter the financial depression again makes itself severely felt, and two intitutions,—the St. Vincent's Hospital and the Melbourne District Nursing Society—are making arrangements to hold balls in aid of their funds. The St. Vincent's Hospital has only been open a little more than half a year, its 35 beds are always full, its outpatients are numbered by hundreds, and its purse is empty. The Nursing Society is endeavouring to maintain three nurses, but it has never received as large a measure of public support as it deserves; apparently the unostentatious nature of its work leaves the public ignorant of the large amount of good it does. Unless the ball returns a substantial amount the usefulness of the society will be seriously crippled this winter. The committees of both balls include the highest names in the land—the Governor and the Countess of Hopetoun, the Chief Justice and Lady Madden, Sir Brian and Lady O'Loghlin, Sir Henry and Lady Wrixom, Sir Frederick and Lady Surgood, Sir B. and Lady Benjamin, the Premier (Mr. J. B. Patterson), the Mayor and Mayoress of Melbourne, and most of the judges, doctors, and consuls and their wives.

Meantime the Committee of the Austin Hospital for Incurables have felt emboldened by a recent increase in funds to reopen the wing for consumptives, and the ceremony was performed on Wednesday, April 26th, by the Mayor of Melbourne (Mr. Arthur Snowden). The building stands apart from the main body of the hospital, and is constructed with spacious rooms and balconies. The largest ward contains five beds; there is a dining-room for men and one for women patients, and the sanitary arrangements are good. The wing has been cleansed and repainted throughout. It is built to accommodate thirty-six—twenty-four men and twelve women. At present the committee, owing to their poverty, are going to take only twelve—eight men and four women—part of the building still remaining closed. It was erected three years ago, and has remained closed for the past nine months. The Austin Hospital is situated at Heidelberg, five miles from Melbourne, and one of the most beautiful spots in Victoria.

Dr. Amy Castilla, who gained the M.B. degree at the end of last year at the Melbourne University and before commencing the five years' course of medicine, had gone through the full course of training—two years—at the Alfred Hospital, as a nurse, has been appointed resident medical officer at the St. Vincent's Hospital.

The case of Dr. Mary Page Stone and the hospitals has excited some attention, though it has not been mentioned in the Melbourne press. The young lady did extremely well during her student time, and at the end of last year came out second in the class list after the examination for the M.B. degree in the second class, there being no firsts. At the beginning of each year the Melbourne Hospital appoints five resident medical officers for a year, and it has always been understood that these positions were reserved for the first five M.B.'s in the class lists. When Dr. Mary Page Stone's name came before the committee, however, that body decided that she was not eligible for a position in the Melbourne Hospital, as she had done all her work in the Alfred—the positions before-mentioned being intended as prizes for the pupils of the Melbourne only. This dictum is now heard of for the first time, and is generally regarded as a mere subterfuge, to get rid of what might have been rather troublesome, the presence of a woman doctor in an overcrowded building. But the point raised by the authorities of the Melbourne is quite likely to be shortly put to the test. Dr. Mary Page Stone was further unfortunate in being rejected at the elections in connection with the Women's and Children's Hospitals, which are both governed by mixed committees, and as it was by the ladies' votes in both cases, it is evident that women are the worst enemies to women's progress.

Dr. Duncan Turner, the author of the little book, "Air and Diet in Chronic Diseases," received his medical education in the schools of Glasgow and Edinburgh.

### Past and Present Workhouses.

The first of a series of articles on "The Nursing and Administration of Provincial Workhouses and Infirmaries" appeared in the *British Medical Journal* of last week. The gradual progress which has been made in London and other large centres since 1865, in which year Mr. Ernest Hart, seconded by Dr. Anstie and Dr. Rogers, instituted an investigation into the condition of sick paupers, is set forth in a most interesting manner. The entire absence of any provision for nursing in those days is partially accounted for by the following description of the Poor Law establishments: "Originally, no doubt, the workhouses were intended for the sturdy vagrant and ne'er-do-well, and partook largely of the nature of prisons; the care of the sick and feeble was an afterthought." Separate wards for the sick, for imbeciles, and infectious cases have been established since then in the London workhouses, also in those in other large towns; but small country workhouses at the present day are often, we are told, in much the same state as the metropolitan ones of twenty years ago.

It is urged that trained nursing should be secured for all sick paupers, and that workhouse infirmaries should be utilised as medical schools. As the writer of the article very justly remarks: "The student might learn the treatment of the chronically sick—a class of patients which represents a large proportion of his practice, and of which, from the nature of his hospital training, he is often very ignorant."

### Nurses as Stewardesses.

In consequence of *The Hospital's* action a few years ago, increasing numbers of trained nurses are employed as stewardesses, greatly to the advantage of travellers. The duties are rather heavy, and include the making up of twenty-five or thirty berths daily. The sheets and coverlets have to be arranged with a mathematical precision which drives the novice nearly to despair. The stewardess begins work, on a big ship at any rate, at a very early hour, and her special department must be ready for inspection, and she herself must appear in spotless uniform, before 11 a.m. The ordinary cases of sea sickness form but a small portion of her responsibilities, which include perhaps half a dozen invalids, some of them really ill. Punctuality, neatness, and accuracy are demanded of the stewardess, and the officers over her make no allowance for any shortcomings in her work. These posts may be recommended as offering many advantages to the competent and conscientious nurse. In spite of her onerous duties she gains greatly in health during a voyage, and on good ships nothing is allowed to interfere with the nurse's two hours off duty daily. The pay is nominally ten or fifteen shillings per week, but much more than this is actually received in fees from her grateful charges. A disposition to make the best of things, and a determination to do her work well, are quite as necessary for a successful stewardess as immunity from sea sickness, and a very large stock of linen aprons, collars, and cuffs!

### English Nurses in Brazil.

(By a Correspondent.)

RIO DE JANIERO.

II.—The Strangers' Hospital (*continued*).

Petropolis is a pretty mountain district about three hours' journey from Rio. Before the revolution, part of the journey was done by crossing the harbour in a ferry-boat, then going up the mountains in the train; now the whole distance has to be accomplished by rail, very tedious at any time, but especially so for business men who do this journey twice daily. In order to escape yellow fever, it is considered a sure safeguard to sleep away from the city during the summer months; so wives and families are packed off up the mountains, and paterfamilias toils up and down every day. There are some perfectly palatial residences, and in all directions right up into the mountains there are evidences of the presence of great wealth. Of course, poor folk cannot afford to live up there, so they have to take their chance in or near the city. So much for the first part of the history of the "Strangers' Hospital."

Of course we did not come as pioneers of English trained nursing without encountering some little drawbacks, so I will just touch upon our first introduction to Rio de Janiero.

We left England at the time of a cholera scare, consequently had to fly the yellow flag, which prevented our entering any of the ports *en route*. We therefore landed at "Ilha Grande," the nearest Brazilian quarantine station to Rio de Janiero, where we were all put ashore to wander about like lost sheep whilst the ship went through the process of disinfection. It was a warm moist day, gentle rain falling and soaking our clothes all through, and when we returned to the ship it was not cheering to find everything drenched with carbolic and water. The poor emigrants had a bad time of it for their bedding was soaking wet. We were not at all sorry to leave this "beautiful isle of the sea" and steam back to Rio, which we did in pouring rain, so there was no landing that day. Next morning dawned beautifully bright, and shortly after breakfast the Rev. Henry Mosely, then president of the Hospital Committee, with another member, came on board to take us off.

We were delighted with the harbour, but our enthusiasm cooled down a little when we got ashore into the hot streets. The first thing that struck us was the very luxurious way in which gentlemen got their boots cleaned. There were rows of huge white umbrellas, under which arm chairs were placed, and in one of these a gentleman can sit and peruse his morning paper whilst his boots are cleaned. The easy way in which people out here take life, and yet get on, is really remarkable. Such a contrast to the rush and wear of the large cities at home.

The hospital not being ready, we were taken to a beautiful place called Tifuca, the "fairy land" of Rio, a fine hotel hidden in a valley surrounded by glorious scenery. Of all the places I ever visited—and these are not a few—nothing has ever compared with beautiful Tifuca. We were installed here for a fortnight, during which time we rambled about, lost in admiration; we also got a little riding, just enough to make us wish for more, but we saw the different views of the harbour from the mountain top, which are very fine. The ferns and palms are marvellously beautiful. There is a place called the "Floresta" here, which may be described as something like Kew Gardens without any glass houses, but everything uncovered and luxurious, and miles and miles of them. Groups of bamboos and grottoes filled with choicest tropical treasures. Such orchids and ferns, cascades, and, in short, everything nature can produce. The place is kept up by the Government.

We soon left this lovely neighbourhood to be nearer to the hospital, in case we should be required. We went to an hotel situated in a very nice part. The house itself seemed all right, but, oh! the dirt of it. The matron had a room on the ground floor off the dining-room, which was infested with cockroaches—not the kind we sometimes meet with in our own dear native land, but huge and startling creatures. Brazil takes pride in producing the largest of their kind in the world. I don't know whether these really were the largest of their kind, but they are much bigger than even Brisbane can produce—three inches in length, at least.

The other Sister and I shared a room at the top of the house, on what was termed the bachelor's landing. We had no cockroaches, but we had an overpowering odour of iodoform. It seemed that a poor fellow had died from yellow fever in that room, and in disinfecting they had been most lavish in the use of iodoform, and the smell clung in a most affectionate manner to the apartment. There was the dirt of ages upon the floor, and many ends of cigarettes. However, we were ready for any sort of experience, and so made the best of it; but dinner-time was the strangest experience. The waiters and cook did not seem on amicable terms, so between the courses they used to retire to the billiard-room, and, judging by the scuffling that went on, personal violence was brought to bear upon whatever question required settling. In the meantime the vegetables on the sideboard grew cold, the cockroaches availed themselves of the opportunity and crawled over them, and this naturally took the edge off our appetites. Dinner over, we generally went for a tram ride, after which to bed, but not always to sleep.

There was an English lady in the hotel who had private rooms nicely furnished. She was most kind; she took us to so many pretty places on the tram, and invited us to her room whenever we liked to go. I should think, during the six weeks we were there, we must have gone hundreds of miles by those trams. The tram service is wonderful, goes everywhere, out along the bays and far into the country; the cars are open and so cool, drawn by mules; it is wonderful how well the little things work, but they seem very well cared for. We also went to the top of the "Corcavado" in the cog-wheel train; it is a fearful journey, over deep ravines on slender, spider-looking bridges, and towards the top it is almost perpendicular, and it is not a nice sensation to know that if so much as a small stone got into the cog wheel the whole train would be wrecked, without any hope of a life being saved. Of course the view from the top is wonderful, and is said to be the most beautiful in the world. It was a lovely clear day when we went, so we saw all to perfection.

You may imagine our delight after six weeks' residence in this place to receive tidings of the hospital being ready for us. We packed up with great zest and bid farewell to cockroaches and iodoform.

Of course the work was not nearly finished for months after we took up our residence at the hospital, but it was nice to feel at home.

Shortly after we went there I was asked to take a private case, a lady who arrived very ill from a steamer, so once more I found myself up at lovely Tifuca, where I stayed six weeks. I had rather a good time during the convalescence of my patient, two ladies staying at the hotel often inviting me to join them in their rides. In this way I saw a great deal of the beauties of the "Floresta." My good fortune seems to follow me, for I have made many kind friends in Rio, as in Australia.

I was recalled to the hospital for the opening, and in the course of the week our first patient arrived. The season was so good there was very little sickness. Of yellow fever we saw but little, most cases entering in the last stage, and dying a few hours after admission; altogether we only had seventeen cases.

Everyone congratulated us upon having no rush of work, saying it would give us time to become acclimatised. So we settled down to a quiet life, most of the cases being intermittent fever, or such medical diseases as heart, lung, or liver troubles.

### Workhouse Infirmary Nursing Association.

ANNUAL MEETING.

From fifty to sixty nurses, with a goodly number of other visitors, assembled on the evening of May 31st at the Nineteenth Century Art Galleries on the occasion of the annual gathering of members of this association. Some very good music filled up the time until the arrival of the President, H.R.H. the Duchess of Teck, who presented medals to the nurses. When this was over, Sir Dyce Duckworth rose to address the nurses. He had had, he said, very large opportunities of watching nursing work, and he was struck, on looking back, by the great difference between the workhouse nursing of to-day and that of thirty years ago, when it was carried on by women who could not be properly called nurses at all, whereas the present workhouse nursing might, he thought, take rank with that in the best hospitals. There were many varieties of the genus nurse, and some were at the present time filled with a spirit of instability and change; but he thought that those nurses succeeded best who found work in some institution and stuck to it. Of course, life in a workhouse infirmary lacked the change and excitement of hospital life; it was inclined to be dull and monotonous, especially in out-lying parts of the country. But those present must remember that they were pioneers in a good work, and such an occasion as the present showed that their efforts were not unappreciated or forgotten. In conclusion he added that the prevailing idea that "they do those things better in France" could not be applied to nursing; in fact, the nursing in French hospitals had deteriorated, not improved, during the last ten years. This was owing to the successive attacks on the Church, resulting in the sisters of mercy (in spite of the expostulations of the medical men) being driven out of the hospitals. Their places had been taken by inadequate nurses, with the result that the patients in some of the hospitals in Paris were worse cared for than in some English workhouses where trained nurses had not been introduced. Miss Louisa Twining, who was very warmly received, said that at their first gathering, fourteen years ago, only five nurses had been present. She wished more could have been present in the room to-night, but it was impossible for many of them to leave their duties. The demand for trained nurses for workhouse infirmaries was greater than the supply at present, and many boards of guardians had applied to them in vain. She was glad to welcome Nurse Hinton, who had so bravely made public the disgraceful state of things at the Newton Abbot Workhouse, and she hoped that this scandal would lead to a thorough investigation of the smaller country workhouses. Very hearty thanks were returned to the Duchess of Teck for her presence on the occasion, and the guests departed, feeling that they had enjoyed an exceptionally pleasant evening.

### Qualified Women Inspectors.

The first lady who presented herself at the Sanitary Institute, Margaret Street, and who passed the examination for Inspector of Nuisances was Miss Scott (now Mrs. Pillow), in December, 1891. In the following year (April, 1892) Miss Lamport successfully followed her example. Since then other ladies have obtained the certificate of the Institute, which declares them to "be competent as regards their sanitary knowledge to discharge the duties of Inspectors of Nuisances." At the examination held in London this spring 61 candidates obtained the certificate, nine of them being ladies, namely, Mrs. C. J. Cassiver, Miss Nora de Chaumont, Miss Annie Duncan, Miss Hannah Elcum, Miss Alice Garbutt, Mrs. Clare Goslett, Miss C. Haynes, Miss Morgan Browne, and Miss Squire. At the examination held in Hull on May 26th, also in connection with the Sanitary Institute, two ladies were amongst the seventeen successful candidates—Miss Margaret Keay and Miss Henrietta Kenealy.

### For Reading to the Sick.

**Motto.**

Cheerfulness has been called the bright weather of the heart. Cheerfulness gives harmony to the soul, and is a perpetual song without words.—*Proverbs.*

**Verses.**

Take joy home,  
And make a place in thy great heart for her,  
And give her time to grow, and cherish her;  
Then will she come and often sing to thee  
When thou art working . . .  
It is a comely fashion to be glad—  
Joy is the grace we say to God. —*J. Ingelow.*

When all Thy mercies, O my God,  
My rising soul surveys,  
Transported with the view I'm lost  
In wonder, love, and praise! . . .  
Ten thousand thousand precious gifts  
My daily thanks employ;  
Nor is the least a cheerful heart  
That tastes those gifts with joy. —*Addison.*

O heart, be comforted,  
And, like a cheerful traveller, take the road,  
Singing beside the hedge! What if the bread  
Be bitter in thine inn, and thou unshod  
To meet the flints? At least it may be said,  
"Because the way is *short*, I thank Thee, God."  
—*E. B. Browning.*

Oh! Lord, unlock my lips in praise,  
By silence chained too long;  
With fervent tune my voice shall raise  
The notes of thankful song.

**Reading.**

Rejoice in the Lord alway, and again I say, Rejoice.—*St. Paul.*

My ills have left me the sun and the moon, fire and water; many friends to pity me; some to relieve me; many tender offices of love, pleasant letters, and some interests and amusements. They have not taken from me, unless I list, my good conscience; they have left me the promises of God, my religion, my hopes of heaven; a soft couch, food to tempt a sick appetite, an airy room and much quiet, and many remedies to try. He that hath so many causes for thankfulness is very much in love with sorrow and peevishness, who loses all these mercies, and chooses to sit down on his little handful of thorns—ever thinking of his ills, and never remembering God's mercies.—*Jeremy Taylor.*

Between levity and cheerfulness there is a wide distinction . . . but it is no unusual thing to betray the one, even when the mind remains an utter stranger to cheerfulness. No doubt it is harder for some natures to be cheerful than for others, yet it is an effort that should be made, for restoration to health is helped by it, and though it seems so difficult at first, it will grow easier the more you practise it.

One great help to cheerfulness is to try to enter into the pleasures of others, and to drive away all envious thoughts of their happiness. Do not suffer them to remain one instant in your heart, but put on a bright face and willingly listen to all that may interest your friends. In the joy of increasing the happiness of others you will receive a blessing yourself. . . . If you turn to Holy Scripture you will learn from St. James an excellent remedy, "Is any among you afflicted, let him pray." Raising up the mind to God in prayer dissipates often the clouds which have arisen, and drives away the evil spirit. You must seek for comfort, and pray for the grace of cheerfulness when you feel oppressed by bitterness of heart. . . . We should look for consolation and encouragement from converse with the Great High Priest, who "can be touched with the feeling of our infirmities, but was in all points tempted like as we are, yet without sin."

St. Chrysostom says, "It is not the nature of the things, but our own dispositions, which usually make us sad or cheerful. If, then, we strive to render our dispositions such as they ought to be, we shall have a pledge for all cheerfulness.

### Where to Go for a Holiday.

### THE PILGRIMS' PATH.

In the days when it was the desire of every Englishman to go on pilgrimage to the shrine of Thomas à Becket, the ways that led to Canterbury were some of the most frequented in the kingdom.

Parts of the path along which the pilgrims went from Rochester to Canterbury are still plainly to be traced; as a rule they seem to have avoided the high roads, going over hill and vale in as straight a line as possible to their goal, often keeping for miles under the shelter of the hills, when there were any, and crossing and recrossing them in order to keep to their straight line. Wherever they left the hills and took to the valleys they planted a yew tree, and again planted another when there was any doubt as to which way they had to go, or when they again returned to the hills.

At the present day the direction of their route is very easily distinguished by these yew trees; to a casual observer they appear dotted down in a promiscuous fashion, but one soon sees that there is method in the seeming disorder, and that these yews were the pilgrims' "sign posts." Fine old trees some of them are, one of the trunks measuring over thirty feet in circumference.

All along the path, for miles on each side of the little village of Boxley, the wild flowers grow in great profusion, and several kinds are to be found there that are seldom met with in other parts of England, all as a rule unusually large and vivid in colour, whilst the scent of the particularly fine wild thyme growing near is a perfume which lives long in the memory.

Sitting by the side of this flower-strewn path under the shade of a pilgrims' yew, a magnificent view is obtained of the great Weald of Kent stretching away as far as the eye can reach. Churches abound near the path, for in the old days many a pious person endowed one as a shelter for the pilgrims, several of whom seem to have found their last resting place in the cool churchyards, having in every sense accomplished their "earthly pilgrimage." In many instances there is no sign of any dwelling near these churches, which shows that they were built entirely for the use of the pilgrims, and in no way to meet any parochial need.

Maidstone makes excellent headquarters for the tourist, the town itself being full of interest and possessing many comfortable old inns.

From Maidstone to Boxley, which is at the centre of this bit of the Pilgrims' Path, is not more than two miles, while from Maidstone about four miles in another direction stands Leeds Castle, the temporary home of many of our kings and queens. It is a remarkable place, and yet few people seem of know of its existence, although its singularity should make it famous.

The castle stands on two islands in the middle of a lake, one part of the building being connected with the other by means of arches, and over these arches run rooms and a corridor.

It has all been rebuilt at a comparatively recent date, the oldest parts that remain being the bridge and the gateway with portcullis and drawbridge, and projecting platform above, from which was poured molten lead, stones, or other missiles upon the heads of the besiegers. The lake covers about sixty acres, and is in the middle of a beautiful park full of grand old trees.

The castle was principally built by Edward I., though there are some parts of it which belong to the eleventh century; it has been the scene of many a dark deed. Jacqueline, of Burgundy, wife of Humphrey, Duke of Gloucester, the "good duke," was burnt as a witch in 1440, and Joan of Navarre, second wife of Henry IV., was also kept a prisoner in this castle. Richard II. started thence on his ill-fated expedition against Ireland, and his queen Isabella received there news of his capture by Henry IV., proceeding to London to obtain a last look at the "discrowned king" on his sad way to the Tower.

The parish church is well worth a visit; it has a massive square greystone tower, which served as a beacon in old days. It also contains a fine peal of bells, and in its churchyard is a yew tree, which shares with one or two others the honour of being "the oldest in England." A mere shell is all that remains of its trunk, and even this shell is divided into two distinct parts; yet the old tree lives on, and seemingly flourishes, for its boughs extend for nearly twenty feet on two sides of it. E. G.

### Exhibitions and Entertainments.

Messrs. Cassell and Co. are holding their twelfth annual exhibition of black and white drawings at Cutlers' Hall, Warwick Lane, Newgate Street. This exhibition is well worth a visit, and repays one the distance one has to traverse to see it, indeed, the hall is not so difficult of access as at first seems. If alone to see the building—the fine entrance hall and the massive carved staircase, any small trouble of getting there is readily forgotten—those who have not seen Cutlers' Hall would be surprised that out of the retiring thoroughfare of Warwick Lane so beautiful a building exists. The pictures themselves hang on the three long screens which monopolise the central part of this spacious hall. Many of them are familiar to us already, through the medium of Messrs. Cassell's various publications, and one is given an opportunity of contrasting the originals and one's memory of the reproductions. Very well for the most part do they stand the test. There are several beautiful specimens of black and white art, conspicuously those from the pencil of Hal Ludlow and G. C. Haité, the latter distinguishing himself, perhaps, the more pre-eminently. In this Mr. Haité is an interpreter of landscape, Dutch scenery being his especial choice. Some of them are most beautiful, as also his English river pieces. As one looks the suggestiveness of his sketches give them colours to our eyes—the meadows seem green, the sky blue—and yet the medium is only that of ordinary black paint. This is due to the poetical mind of the artist, it is not alone due to the observer that Mr. Haité's landscapes seems painted in varied colouring. Amongst the four hundred and forty-two pictures, there are many originals of artists whose names are perhaps better known. For instance, MacWhirter, Arthur Hopkins, Alfred East, and W. L. Wyllie. The exhibition is open for a short time only—from May 31st to June 15th.

Meeting of the Parents' National Education Union, 28, Victoria Street, S.W., at 4 p.m., on Friday, June 8th.

Baby's Exhibition at Humphrey's Hall, Knightsbridge, from June 2nd to 29th. Mrs. Ballin, editor of the magazine called *Baby*, will lecture on all Mondays in June on "The Clothing of Infants and Children"; on Tuesdays, on their "Feeding"; on Wednesdays, on "Exercise and Rest"; on Thursdays, on "Education"; and on Fridays the subjects will be "Common Complaints: Their Prevention and Cure."

Lecture at Trained Nurses' Club, 12, Buckingham Street, on Friday, June 29th, at 7.45, Dr. R. S. Risien Russell on "Disorders of the Nervous System met with in the Course of other Diseases."

Bolingbroke Hospital, Wandsworth Common.—On Saturday, June 16th, a garden fête and sale of work will be held in the grounds on behalf of the General Fund of the hospital. Gifts of work and contributions towards the refreshments will be gladly received by the Matron.

Nurses' Co-operation, 8, New Cavendish Street.—On June 13th, at half-past three p.m., a general meeting will be held of the committee and nurses of the Co-operation.

### The Nurse Dolls.

The Nurse Dolls were greatly appreciated during their recent visit to Hull, where they assisted at a bazaar held in aid of the Children's Hospital. The bazaar was a most successful one, and we are told that the dolls "looked very nice," and had clean aprons and caps for the occasion.

☞ For The Muses' Looking Glass, Everybody's Opinion, Book World for Women and Nurses, &c., see page xcix. et seq.

### The Muses' Looking Glass.

### "FAIR WOMEN" AT THE GRAFTON GALLERY.

Few exhibitions of late years have excited so much attention as the above—the very novelty alone of the scheme having an attraction to the public. Beautiful women of all ages and climes in manifold portraits adorn the walls on all sides, represented in all their varied phases—the child with laughing eyes—the Court beauty—the crowned head. Some exception has been taken to the title of the exhibition as there are included certain pictures of women possibly more celebrated for their historical interest or their wit than perhaps for their actual form. The directors explain, however, that "they do not know of any fixed standard by which such pictures can be judged, and, further, they believe that in the eyes of some one person, almost every woman has been considered fair."

The first three galleries have been arranged with a general idea of chronological sequence, and the first portrait which catches one's eye upon entering is of a lady of the second century A.D. The perfect preservation of this is a little astonishing considering its antiquity. On the other side of the wall a companion picture hangs, painted at the same period. Both these curious pictures were taken from mummy cases found in Egypt. They are worked in tempera, and show ladies with no mean claim to physical beauty. There are no specimens of early art between these of the second century, and some of Botticelli's and Hans Holbeins representing a gap of twelve or thirteen hundred years. The portrait of a lady by Piero Della Francesa (No. 3), a product of 1400, is a composition of paint and needlework, the bodice of the model's gown being elaborately stitched in many colours.

In the Music Room we meet with many familiar faces from the brushes of Rubens, Van Dyck, Peter Lely, Hoffner, Reynolds, and Romney. If for this room alone the gallery is worth many a visit. Scattered here and there among these are some exquisite specimens of Greuze's work—bewitching child-like faces, devoid of guile with parted lips, and eyes which meet one with their straight direct gaze. There are few lovelier pictures here than these, fascinating one as they do with their perfect simplicity. It is not given to every painter to depict the innocent beauty of childhood. Greuze is a sublime master of the art, and the freshness of his fair faces haunt one long after one has passed them by.

Sir Joshua Reynolds and Romney are seen pretty much at their best. One—hung side by side with Gainsborough's "Lady Hamilton as Ariadne"—by Romney is especially charming. Of this beauty there are numerous portraits throughout the gallery, but none show her to more advantage than this particular one.

Another picture which attracts a great deal of attention, and which hangs almost next to the one we have just referred to, is a life-size portrait of Mrs. Michael Angelo Taylor, as "Mirauda," by John Hoppner. The figure and face are both striking, as is also the artistic manner in which they are treated. Mrs. Taylor is clad in the lightest of light raiment, and her feet are shod in tiny white satin slippers. This has its inconsistent side, considering the background of stormy skies and raging seas. The lady herself is on the surf, and the winds are playing havoc with her skirts and her curls. There was a time—and it is perhaps as well that it has passed—when storms were considered as necessary to the background of portraits as in earlier times were the cherub's faces which they superseded. If they were necessary, they were none the less incongruous, and more especially so in the case of the fine picture we have referred to above. Nearly all the canvas in this Music Room deserve individual notice did space permit.

The Centre Room, though containing many old masters, lands us in modern times again, amongst many pictures, too, which we readily recognise. Here is a fine portrait by Von Angeli of the Queen, resplendent in Crown jewels. Jean Boldine's canvas of Lady Colin Campbell is very spirited and striking, and has a unique charm, foreign to its surroundings.

There are two canvasses of Greuze which cannot be passed by without some comment, these are the portraits of Cecile Volage (106), and Madame du Barry (104). The former shows us a face of rare and exceeding loveliness. Once upon a time this fair French girl was the beauty of her day—and Greuze shows her to us in all the radiance of her youth. It is not alone in form of features that the charm of Mademoiselle Cecile lies, for the gentle, sweet expression of eyes and mouth have as great a fascination.

And side by side with this picture hangs the portrait of poor Madame du Barry. It may be one's imagination, but there seems even now in the lady's eyes a pathetic appeal for mercy, as if already she felt the doom of the approaching guillotine was looming near.

A large central canvas of Ellen Terry, by John S. Sargent (No. 154), is both impressive and dramatic. The actress is represented as Lady Macbeth, and besides being a fine picture, it has the further merit of being a good portrait. Near by is Professor Herkommer's famous study of Miss Grant—by the side of which this artist's later works, as exhibited at the Academy and the New Gallery, contrasts but poorly. Long ago, Sir John Millias' portrait of Miss Eveleen Tennant was one of the pictures of the year—that is hanging here now (No. 150).

So much for the pictures, of which more cannot here be particularised.

Of the miniatures, of which a great number are exhibited, it would be hard to say sufficient, so beautiful and so priceless are they. Nearly all the Court beauties and many crowned heads are here to be found, Lady Hamilton and Mary Queen of Scots again being conspicuous. Madame de Pompadour, too, Marie Antoinette, and the Empress Marie Louise of Austria, immortalised on her husband's snuff-box. Of course, the Cosways possess the greatest interest, and stand the test well of all contrast in their surroundings. There are some exquisite examples of the artist's work. The miniature of a lady unknown is one among the many of these which rivet our attention. Indeed, such a galaxy of beauty as we wander through in the comparatively confined space of the galleries makes the every-day world of ordinary beings appear sadly unbeautiful in the contrast!

Another great interest in the exhibition are the curious relics of dead and bygone women. Long cases down the galleries enclose these in all their multiform varieties. Patch boxes, powder boxes, rouge pots, dressing cases, knick-knacks, jewels—the hundred and one accessories of the female toilet. Dresses, too, and costly lace, Court robes, riding jackets, and Queen Elizabeth's shoes side by side with her violin. Such a clumsy violin as it is, carved and heavy, ponderous as a 'cello in its massive proportions. And here, too, is the watch of good Queen Bess, and the heavy chatelaine from which it hung suspended.

Not very far off these lie the relics of her less fortunate cousin, the luckless Mary Queen of Scots. The very ornaments worn by that unhappy woman on the day of her execution at Fotheringay Castle are here displayed; and most pathetically they appeal to us even at this distance of time. Indeed, the past is made very present to us as we wander through these galleries. Some, indeed, of the beautiful faces whose likenesses adorn the walls of the Grafton Galleries are alive, and grace the world of to-day, but the greater number have long passed away from us, and only their portraits and their belongings remain to remind us of those other fair women who once existed.

### Everybody's Opinion.

ESSEX COUNTY COTTAGE NURSING ASSOCIATION.

Dr. J. C. Thresh, Honorary Secretary, writes: I note that you occasionally honour us with a notice. Would it not be well for the reputation of your journal to make sure of your facts before offering criticism. It has never once been suggested by any member of this association that women who had only had three months' training should be sent out as nurses, either into cottages or elsewhere.

*** Dr. Thresh encloses a printed statement of the qualifications and selection of candidates for training at the expense of the association by a sub-committee consisting of six medical men, Miss Luard and Sister Katherine Twining. Dr. Thresh marks rules three and five which are as follows:—

III. She must produce evidence satisfactory to the committee that she has already been accustomed to act as a nurse, and that she has shown special aptitude for nursing, although untrained.

V. The Executive Committee shall select such of the candidates as are, in their opinion, most fitted to assist in the work of the association, and shall decide upon the length and character of the course of training which each shall undergo.

Can it be possible that Dr. Thresh has forgotten his statement as reported in the *Colchester Standard* of May 19th last, that "they would be able to train six probationers at a time," who "would stay either three or six months"?—Ed. *T. H.*

DISTRICT NURSING.

"One Who is Interested" writes: The article in your paper on "District Nursing" has only just come under my notice, but as one for many years very intimately connected with, although not actually engaged in, nursing, may I be allowed to warmly support the other side of the question? A thoroughly-trained nurse, who loved her work, would not be content to get out of practise by taking district work in a country place, while there are many women having had short hospital training and (often) long other experience, with good common-sense (which does not of necessity accompany three years' hospital training), love for work, and quite capable of an intelligent obedience to the doctor's orders, who would be content to take those posts at half the cost the more highly-trained ones would require, but whose skill would so rarely be called into exercise. As a rule it is my experience, both in infirmary and nursing institutions, that not the lady is sought for as making the best nurse, but the upper-class servant; nor would the practical "heart in her work nurse" find time or care for carriage drives, except as help in long distances, and even then a friendly passing cart would answer best. Certainly the decision of the poor is in favour of those nearest their own station, who in cases of necessity would not scruple (duty allowing) to lend a hand in little services, not strictly nursing, but indirectly of importance in their effect on the patient's health. However, I write to protest against the exclusion of sensible, practical women from the post of district nurse, at all events in country places, because they may not have had a full three years' training, and also to claim their right to the title of nurse, though not of trained nurse.

*** The writer of this letter is evidently, as she says, "not actually engaged in nursing," and therefore she must be excused for having taken a superficial view of her subject. As a district nurse must add training in district work to her hospital experience, she is not likely to consider that nursing the sick poor in their own homes will let her "get out of practice." On the contrary, her knowledge will increase with her experience. "Little services, not strictly nursing, but indirectly of importance in their effect on the patient's health," would certainly always be considered by the well-trained nurse as lying within her province. It is generally an inexperienced probationer, or one of those "with short hospital training," who designates these "little services" as "menial." The upper class servant is acknowledged to possess many of the essentials of a first-rate nurse when she has received full training, and few of these intelligent young women would be contented with less. We cannot see why a nurse, "with her heart in her work," should prefer "a friendly passing cart" to a carriage. We do not wish to disparage the former vehicle, but are strongly in favour of the latter, where possible, for a hard-working nurse, who certainly merits every facility for rest and ease in the intervals of duty. Far from wishing to exclude "sensible practical women" from the posts of district nurses, we only wish there were more of them, and their value will be largely enhanced when they receive the complete training, which is, we maintain, absolutely necessary for them.

NOTICE TO CORRESPONDENTS.

"Queen's Nurse" is informed that her communication has been carefully considered, and that full inquiries have been made into all the circumstances referred to. We are of opinion that she would be well advised in her own interest, seeing that she is wrong in her facts and labours under misapprehension on several points, if she would take herself to task a little, and try to be more conciliatory and less wedded to her own opinion when dealing with patients, doctors, and those with whom she is brought in contact.

### A Successful Café Chantant.

On June 2nd a large company assembled at the Portman Rooms for the Café Chantant of the Gordon Boys' Home. The entertainment was a financial success, and the programme was of a most attractive character. The string band of the Royal Marines and the Ladies' Guitar, Mandoline, and Harp Band added greatly to the enjoyment of those present. Mademoiselle Lanari, Miss Esther Palliser (who sang a new song, accompanied by the composer, Miss Eva Lonsdale), Madame McKenzie (who also sang a charming new song by Mrs. Edith Dick), Mr. Barrington Foote, Miss Cissie Loftus, and others appeared on a programme which brought together a most influential and fashionable assembly to support a very good cause.

### Appointments.

Cumberland and Westmoreland Counties Asylum.—Miss Maria Robinson has been appointed Matron at this hospital. She was trained at Mavistack Bank Private Asylum, the Perth District Asylum, Murthly, and held the post of Matron and Assistant Matron at the Inverness District Asylum. Such large experience specially qualifies Miss Robinson for a position in which we wish her every success.

Kendal Memorial Hospital.—Miss Janet Lindsay, who was trained at the Liverpool Infirmary, has been made Matron of the Kendal Memorial Hospital. She takes many good wishes with her to her new post.

### Minor Appointments.

Poplar Hospital for Accidents.—Miss Blackett has been appointed Night Sister at this hospital. She was trained at the London Hospital, where she remained three and a half years, and for two and a half years held the post of Nurse at the Hospital for Women, Chelsea. We wish Miss Blackett success in her new work.

### Notes and Queries.

**Queries.**

(76) *Information.*—Can you tell me where probationers are taken for training, Children's or Infectious Hospital preferred?—*G. R. G.*

(77) *Queen's Nurses.*—Where can I get information about training for a Queen's Nurse?—*P. G.*

(78) *Fumigation.*—How can I keep a room clean after it has been stoved?—*Subscriber.*

(79) *Roman Catholic.*—Can I obtain nursing work through any of the religious orders in England?—*Nurse B.*

(80) *Midwifery.*—Information as to training in monthly nursing, &c., wanted by *A. E. C.*

**Answers.**

(76) *Information* (*G. R. G.*)—Write to the matrons of the principal children's hospitals and ask for the rules and other particulars. You will find the institutions in "Burdett's Hospital Annual." You should read "How to Become a Nurse" by Honnor Morten. Both these books are published by the Scientific Press, 428, Strand, London.

(77) *Queen's Nurses* (*P. G.*).—Write to Miss Peter, Inspector, Q.V.J.I., at St. Katharine's Hospital, Regent's Park, London, for all particulars.

(78) *Fumigation* (*Subscriber*).—If the room has been thoroughly done with sulphur it should need nothing further than ordinary washing of walls and floor. If you use carbolic soap, take care to get it strong and good.

(79) *Roman Catholic* (*Nurse B*).—Inquire of the Superior at Nazareth House, Hammersmith.

(80) *Midwifery* (*A. E. C.*)—Address the Hon. Secretary of the Workhouse Nursing Association, 6, Adam Street, Adelphi, Strand.

### The Book World for Women and Nurses.

[We invite Correspondence, Criticism, Enquiries, and Notes on Books likely to interest Women and Nurses. Address, Editor, The Hospital (Nurses' Book World), 428, Strand, W.C.]

A Protégée of Jack Hamlin's. One vol. By Bret Harte. (London: Chatto and Windus, 1894.)

The title of this book is that of the first of six short stories published together in one volume. Each of them is furnished with at least one suitable illustration, and there is a frontispiece—a portrait of Jack Hamlin. This latter, we fear, will be as great a disappointment to his admirers as the actual appearance of a celebrity generally is. It is certainly not one's mental vision of the hero of "The Outcasts of Poker Flat." By his side in the picture is his protégée, a young woman whom he rescues from suicide and adopts temporarily as his niece. All our previous impressions of Jack Hamlin's chivalry and delicacy towards women are justified by his treatment of the girl. There was no question of love between them. To Jack she was nothing but an ordinary, rather uninteresting young person in all but that she had sufficient desperation in her nature to attempt to take her own life, and this desperation appeals to his strongest feelings and at once enlists his sympathy. If a fault may be found with a story so charmingly told, it is that it is just a little too melodramatic, and Jack Hamlin takes us a little aback when he sits down to an American organ and plays an "Agnus Dei." Of the other stories "The Ingénue of the Sierras" is by far the best, and the drawing of the Ingénue herself is an artistic triumph. She is a grand example of how a woman at bay can save an almost impossible situation by generous lying, provided it be done with sufficient candour and unscrupulousness—characteristics in which no self-respecting heroine of Mr. Bret Harte has ever been found wanting. Our old friend Yuba Bill figures in this story, and the Ingenue fulfils a difficult mission when she succeeds in making a complete fool of him. Taken as a whole, the author has shown himself quite up to his old inimitable form in the writing of these six tales.

St. Ann's. By W. E. Norris. 2 vols. (London: Chatto and Windus, 1894.

A new novel from Mr. Norris always meets with a well deserved welcome, and "St. Ann's" fully justifies our expectations. The plot is neither very striking nor very original, but the interest is well sustained and the story charmingly told in Mr. Norris's gentlemanly manner, if one may apply the adjective in a literary sense. The scene is laid in Devonshire, where Arthur Foley, the hero of the story, and his father are spending the summer months. Young Foley distinguishes himself by falling in love with two young women at the same time. In neither case does his choice altogether enlist our sympathy. Miss Rhoda Meynell, however, the lady on whom he ought to have concentrated his affections, is obviously all-sufficing in the eyes of the author. She is a heroine after Mr. Norris's own heart. Yet refined, intelligent, self-controlled, and pretty as she is, her very virtues are a little annoying, and it is hardly to be wondered at, if her self-control finally appeared unfeeling in her lover's eyes. Then her blind obedience to her stepbrother is a trifle exasperating, although a not unusual virtue in the female relations of retired colonels with a turn for *al fresco* preaching. We have a lurking sympathy with Arthur, when, on being refused the hand of Rhoda by Colonel Meynell, who thinks it unnecessary to consult his sister, he gets caught in the toils of his beautiful cousin, Lola Hammersley. Everyone now seems about to marry the wrong person, and Mr. Hammersley emphasizes his objections to the match between Arthur and Lola almost to the point of murder. Here, however, retributive justice steps in, a gun goes off accidentally—and a bullet in Mr. Hammersley's right lung silences his objections for a time. It would be a pity to spoil the story for future readers by revealing how everything comes right in the end—suffice it to say that "St. Ann's," is a delightful story, and no unworthy successor to "A Batchelor's Blunder," and "My Friend Jim."

The Theory and Practice of Laundry Work for Scholars. Mrs. E. Lord. (London: Joseph Hughes and Co. 1894.)

Mrs. Lord, who is senior organizer and Superintendent of Laundry to the London School Board, speaks with the authority of much experience in the small text-book on laundry work for young scholars which is before us. Concise and simple information on practical points connected with the work of the laundry, in book form, will no doubt be found of much use in a wider sphere than that for which it is primarily intended, and the instructions are given in a clear manner which adds much to their value. Even though steam laundries are now being so largely introduced, a thorough knowledge of "hand-washing" can never be superfluous, and is rightly recognised as an important branch of technical education for girls. Mrs. Lord takes her scholars through the week's work, starting with a programme of how the business in hand can be best arranged, and a consideration of the materials to be used. Some invaluable hints on the removal of stains, &c., are contained in Chapter II. Details of ironing, folding, disinfecting, and the treatment of "fine things," &c., are thoroughly gone into; in fact, the subject is treated on the whole most comprehensively. Some of the information may be a little over the heads of very juvenile learners, but Mrs Lord explains in a short preface that this is designed to form part of a more extended course of laundry work to follow in due time. The little book is attractively got up and illustrated.

### THE REVIEWS.

The Contemporary Review, in an article with the somewhat sensational title of "Halt," advocates, as a first step towards national disarmament, an agreement among the Powers on the maximum amount which they are to spend on their respective armaments for the next six years, until the opening of the twentieth century. The monetary check, says the anonymous author, is not only the simplest but would involve the least interference in the internal administration of each state by its partners in the bond. It is comparatively useless, he tells us, to try to limit the number of men—and certainly Napoleon failed in the attempt, even with Prussia abject at his feet. Further, all the powers of Europe are longing for some such escape from their self-imposed burdens, and even France, the only stumbling block in the pathway of peace, would be glad to hearken to overtures on the subject, provided only that they did not emanate from the arch-enemy at Berlin.

Miss March Phillips' contribution on the new Factory Bill and its effects upon women, in the Fortnightly Review, is a paper that everyone interested in women's work should make a point of reading. The picture she draws of the trades at which women are working for starvation wages, not only amidst hardship and discomfort but amidst positive dangers to health and life, is not one to be easily forgotten. A good deal has been said of the unwillingness, especially of the women engaged in laundry work, to be "interfered" with as regards the conditions of their labour. According to Miss Phillips this unwillingness has been much exaggerated, and the great majority of laundresses are looking eagerly forward to the protection such an Act as Mr. Asquith's would afford them. Perhaps the best political article of the month is also to be found in the pages of the Fortnightly—Mr. Wallace's forecast of "The Future of Parties."

The frontispiece of Scribner's Magazine, Mr. Stanhope Forbe's "Lighthouse," is accompanied by a short sketch of the painter's life and work from the hand of Mr. P. G. Hamerton. Mr. J. Heard's "Maxmilian and Mexico" is interesting, not only as an account of the short and troubled reign of the hapless Emperor-Archduke, but also for the idea pervading that crowned heads and tyranny are things inseparable, which crops up here and there in delightfully republican expressions.

